# Reproducibility of thoracic kyphosis measurements in patients with adolescent idiopathic scoliosis

**DOI:** 10.1186/s13013-017-0112-4

**Published:** 2017-02-21

**Authors:** Søren Ohrt-Nissen, Jason Pui Yin Cheung, Dennis Winge Hallager, Martin Gehrchen, Kenny Kwan, Benny Dahl, Kenneth M. C. Cheung, Dino Samartzis

**Affiliations:** 1 Spine Unit, Department of Orthopedic Surgery, Rigshospitalet, University of Copenhagen, Blegdamsvej 9, Copenhagen East, 2100 Denmark; 2 Department of Orthopedics and Traumatology, The University of Hong Kong, Professorial Block, 5th Floor 102 Pokfulam Road, Hong Kong, SAR China

**Keywords:** Adolescent idiopathic scoliosis, Thoracic, Kyphosis, Radiograph, Sagittal, Flexibility, Reproducibility, Reliability, Agreement, Intra-class correlation, Mixed effects model, Repeatability coefficient, Limits of agreement

## Abstract

**Background:**

Current surgical treatment for adolescent idiopathic scoliosis (AIS) involves correction in both the coronal and sagittal plane, and thorough assessment of these parameters is essential for evaluation of surgical results. However, various definitions of thoracic kyphosis (TK) have been proposed, and the intra- and inter-rater reproducibility of these measures has not been determined. As such, the purpose of the current study was to determine the intra- and inter-rater reproducibility of several TK measurements used in the assessment of AIS.

**Methods:**

Twenty patients (90% females) surgically treated for AIS with alternate-level pedicle screw fixation were included in the study. Three raters independently evaluated pre- and postoperative standing lateral plain radiographs. For each radiograph, several definitions of TK were measured as well as L1–S1 and nonfixed lumbar lordosis. All variables were measured twice 14 days apart, and a mixed effects model was used to determine the repeatability coefficient (RC), which is a measure of the agreement between repeated measurements. Also, the intra- and inter-rater intra-class correlation coefficient (ICC) was determined as a measure of reliability.

**Results:**

Preoperative median Cobb angle was 58° (range 41°–86°), and median surgical curve correction was 68% (range 49–87%). Overall intra-rater RC was highest for T2–T12 and nonfixed TK (11°) and lowest for T4–T12 and T5–T12 (8°). Inter-rater RC was highest for T1–T12, T1-nonfixed, and nonfixed TK (13°) and lowest for T5–T12 (9°). Agreement varied substantially between pre- and postoperative radiographs. Inter-rater ICC was highest for T4–T12 (0.92; 95% CI 0.88–0.95) and T5–T12 (0.92; 95% CI 0.88–0.95) and lowest for T1-nonfixed (0.80; 95% CI 0.72–0.88).

**Conclusions:**

Considerable variation for all TK measurements was noted. Intra- and inter-rater reproducibility was best for T4–T12 and T5–T12. Future studies should consider adopting a relevant minimum difference as a limit for true change in TK.

**Electronic supplementary material:**

The online version of this article (doi:10.1186/s13013-017-0112-4) contains supplementary material, which is available to authorized users.

## Background

Adolescent idiopathic scoliosis (AIS) is characterized by a lateral deviation of the spine in the coronal plane, vertebral rotation in the transverse plane, and often hypokyphosis in the sagittal plane [1, 2]. Current surgical treatment for AIS involves multisegmental pedicle screw instrumentation, which results in considerable correction in the coronal plane with limited loss of correction over time [3, 4]. However, several studies have reported failure to restore the thoracic kyphosis (TK) to a normal range seen in non-scoliotic subjects, and in recent years, the importance of surgical correction of sagittal malalignment has gained increased focus [5, 6].

Although measuring TK in AIS patients on plain radiographs has become commonplace throughout the decades, considerable variation across studies in terms of defining TK exists and no consensus has been established on what should be regarded as an actual change in TK as opposed to expected measurement variation. For one, a recent meta-analysis evaluated the surgical correction of TK in AIS patients; however, the analysis included various studies with different definitions of TK, which made direct comparisons challenging [7]. Moreover, several studies have attempted to define the TK range in normal subjects but have used different definitions without addressing differences in measurement variation [8–10]. Furthermore, the Lenke classification [11] is widely used in preoperative planning; however, classification of the sagittal thoracic modifier has shown poor reliability and the measurement agreement for T2–T12 and T5–T12 kyphosis have been found to be reduced compared to the frontal Cobb angle [12, 13]. For T2–T5 regional kyphosis, reliability has been shown to be poor [13, 14] and other studies have shown that the upper part of the thoracic spine is inherently challenging to visualize due to structural overlap of the shoulder girdle [8, 15, 16]. As the clinical importance of the spinal sagittal profile becomes increasingly evident, there is a need to ensure that the measuring methods used to evaluate TK are both accurate and reproducible, especially since the rotational component of the curve may alter reproducibility depending on definitions of TK. Traditionally, TK is determined by a fixed limit Cobb technique (fixed TK, e.g., T4–T12); conversely, the definitions of fixed TK vary among studies [17–20]. A few authors have suggested applying a nonfixed approach where limits of TK are based on the individual sagittal shape of the spine as it has been shown that the cranial and caudal end vertebrae of the nonfixed TK vary among normal adolescents [8, 21–23].

Overall, the intra- and inter-rater reproducibility of these various TK measurements has not been established, and there is no consensus as to which measurements offer the least amount of variability. While a few studies have addressed the intra- and inter-rater correlation for certain TK measurements [24], it is of limited application on individual patients and it will be of great clinical and academic value to know the actual expected variation for repeated TK measurements on the same subject. As such, the objective of the following study was to determine intra- and inter-rater reproducibility of commonly used TK measurements.

## Methods

Plain radiographs of 20 patients who were at one point diagnosed with AIS and surgically treated at our institution with alternate-level pedicle screw fixation [25, 26] were examined. Institutional review board approval was obtained. Gender and patient age was recorded, and curve type was determined based on the Lenke classification [27]. On the coronal radiograph, pre- and postoperative main Cobb angle was measured and correction rate was calculated.

**Fig. 1:**
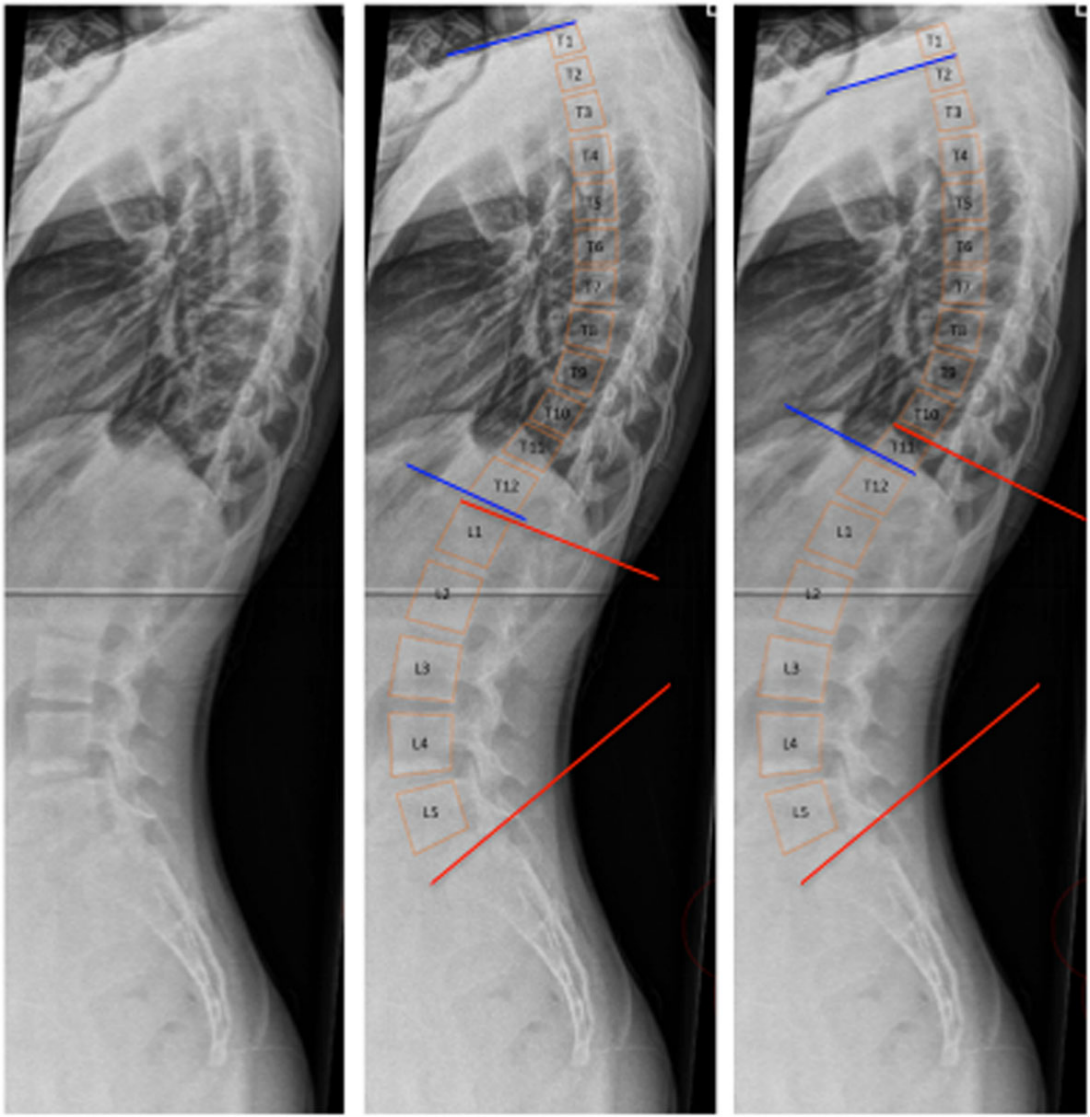
*Left*: Standing sagittal radiograph of a thoracic single curve with apex at T8. *Middle*: Fixed measurements of T1–T12 thoracic kyphosis (*blue*) and L1-S1 (red). *Right*: Nonfixed measurements of thoracic kyphosis (*blue*) and lumbar lordosis (*red*)

One spine research fellow (rater 1) and two spine surgeons (raters 2 and 3) independently evaluated 20 sets of pre- and postoperative standing lateral radiographs. For each radiograph, the following were determined (Fig. 1):

1. Fixed TK defined as the Cobb angle between the superior end plate and the inferior end plate of T1–T12, T2–T12, T4–T12, and T5–T12 [28]
2. T1-nonfixed TK: From the superior end plate of T1 to the inferior end plate of the most tilted vertebra in the thoracolumbar region [21, 22]
3. Nonfixed TK: From the superior end plate of the most tilted vertebra in the proximal thoracic region to the inferior end plate of the most tilted vertebra in the thoracolumbar region [8]
4. Fixed lumbar lordosis (LL): From the superior end plate of L1 to the superior end plate of S1 [29]
5. Nonfixed LL: From the superior end plate of the most tilted vertebra in the thoracolumbar region to the superior end plate of S1 [23]

Each rater independently performed all measurements twice 14 days apart. Before the second round of measurements, the sequence of the radiographs was randomly reassigned and the raters were blinded from the results of the first round. All raters were blinded to patient details. The total analysis produced 1920 data points for further analysis. All radiographs were measured on a high-resolution monitor using the Picture Archiving Communication system, and identification and labeling of individual vertebrae was based on the Radiographic Measurement Manual by the Spine Deformity Study Group [28]. Application of this manual was discussed among the raters, and consensus was established prior to the study. The protocol for the study was based on the Guidelines for Reporting Reliability and Agreement studies [30].

### Imaging details

For the scoliosis radiographs, all patients were positioned in erect position with the feet together and in the straightest posture possible. For lateral images, patients were in the clavicle position with flexed shoulders and elbows past 90° with hands pointing at the sternal notch to allow better spine visualization while preventing changes to the sagittal balance [31]. A computed detector was utilized to determine the position of the patient’s skull and hip joints and also the length of the image required. The detector was 40 cm in length, and thus, image splitting was required. Up to 2–3 exposures were required depending on the patient’s height. The postero-anterior radiographs were taken with 78-peak kilovoltage and 20 mAs of X-ray energy. The lateral radiographs were taken with 88-peak kilovoltage and 32 mAs of X-ray energy. For both images, the focus film distance was 180 cm.

### Statistical analysis

All statistical analyses were performed using R version 3.2.3 (R core team, 2014, Vienna, Austria). Data was reported as proportions (%), mean with standard deviation (SD), or median with range, and data distribution was assessed by histograms.

Reproducibility is a term that entails both measurement agreement and reliability. Intra- and inter-rater agreement is defined as the degree to which repeated measurements are identical whereas reliability is defined as the ability of a measurement to differentiate between subjects [30]. Intra- and inter-rater agreement per subject was estimated for each type of TK measurement using the repeatability coefficient (RC), which is the difference in measurements exceeded by only 5% of pairs of measurements on the same subject. Ninety-five percent limits of agreement were defined as ±RC, meaning that a high RC indicated a high variation (poor agreement) in repeated measurements.

Intra-rater agreement for each rater was calculated according to Bland and Altman [32]:

- Single rater RC = 1.96 * SD of the difference between repeated measurements for each rater.

Overall, intra- and inter-rater RC was calculated using a linear mixed effects model with subjects and rater-within-subject variation as random effects and timing of radiograph (e.g., pre- or postoperative) as a fixed effect: [24]

- Overall intra-rater RC = 2.77 * √(residual mean square)
- Overall inter-rater RC = 2.77 * √(rater:subject mean square + residual mean square)

Inter-rater RC was further analyzed for pre- and postoperative radiographs separately.

Intra- and inter-rater reliability was estimated with intra-class correlation coefficient (ICC) with 95% confidence interval (CI). We considered an ICC of 0.0–0.24 to represent absent to poor, 0.25–0.49 low, 0.50–0.69 fair/moderate, 0.70–0.89 good, and 0.90–1.0 excellent reliability [33, 34].

## Results

Eighteen patients were female (90%), and the median age was 13.8 years (range 11.5–27.6 years). Eighty-five percent of curves were Lenke type 1 and 15% Lenke type 3, and the preoperative median coronal Cobb angle was 58° (range 41°–86°), which was corrected to a postoperative median Cobb angle of 20° (range 8°–27°) corresponding to a median curve correction of 68% (range 49–87%). Median number of fused levels was 9 (range 6–11 levels). The upper instrumented vertebra was T4, T5, T6, and T7 in one, 13, five, and one patient, respectively. Lowest instrumented vertebra (LIV) was T11, T12, L1, L2, and L3 in two, five, eight, three, and two patients, respectively. Summary of all measurements of both pre- and postoperative radiographs for each round is listed in Table 1 (Additional file 1).

**Table 1:** Summary of measurements for each rater for pre- and postoperative sagittal radiographs of both rounds of measurements

Variable	Rater 1 Cobb angle, mean ± SD		Rater 2 Cobb angle, mean ± SD		Rater 3 Cobb angle, mean ± SD	
	Round 1	Round 2	Round 1	Round 2	Round 1	Round 2
T1–T12						
Preoperative	29 ± 11	29 ± 11	30 ± 11	32 ± 10	34 ± 9	31 ± 10
Postoperative	26 ± 7	26 ± 8	30 ± 9	32 ± 8	31 ± 6	31 ± 7
T2–T12						
Preoperative	28 ± 12	28 ± 12	31 ± 13	30 ± 10	33 ± 14	28 ± 11
Postoperative	26 ± 8	26 ± 9	29 ± 8	31 ± 8	28 ± 6	28 ± 8
T4–T12						
Preoperative	24 ± 13	23 ± 12	26 ± 13	25 ± 12	29 ± 15	26 ± 13
Postoperative	19 ± 7	18 ± 6	21 ± 8	19 ± 7	21 ± 6	20 ± 6
T5–T12						
Preoperative	23 ± 12	21 ± 12	25 ± 12	23 ± 13	25 ± 14	24 ± 14
Postoperative	16 (6)	16 ± 7	17 ± 7	16 ± 6	18 ± 6	18 ± 6
T1-nonfixed						
Preoperative	30 ± 12	30 ± 11	30 ± 12	32 ± 10	35 ± 10	32 ± 11
Postoperative	27 ± 7	27 ± 8	31 ± 9	32 ± 7	32 ± 7	31 ± 7
Nonfixed TK						
Preoperative	32 ± 11	31 ± 11	35 ± 12	35 ± 10	37 ± 11	34 ± 13
Postoperative	28 ± 8	28 ± 9	32 ± 10	34 ± 9	33 ± 7	32 ± 8
L1–S1						
Preoperative	58 ± 11	56 ± 10	55 ± 11	58 ± 12	55 ± 10	56 ± 10
Postoperative	55 ± 8	53 ± 9	51 ± 9	53 ± 9	49 ± 9	52 ± 8
Nonfixed LL						
Preoperative	60 ± 11	60 ± 11	60 ± 12	62 ± 12	58 ± 12	59 ± 11
Postoperative	57 ± 9	55 ± 9	55 ± 9	57 ± 9	52 ± 9	54 ± 9

*SD* standard deviation, *TK* thoracic kyphosis, *LL* lumbar lordosis

### Intra- and inter-rater agreement

Single rater RC showed substantial differences among raters ranging from 5° to 13° (Table 2). Overall intra-rater RC was highest for T2–T12, T1-nonfixed, and nonfixed TK (11°) and lowest for T4–T12 and T5–T12 (8°). The overall inter-rater RC was highest for T1–T12, T1–nonfixed, and nonfixed TK (13°) and lowest for T5–T12 (9°) (Table 3). Inter-rater RC ranged between 7° and 14° across pre- and postoperative radiographs. For fixed LL and nonfixed LL, variation was similar to intra- and inter-rater RC ranging from 10° to 11° (Tables 2 and 3).

**Table 2:** Single rater RC for all three raters and overall intra-rater RC with pre- and postoperative subgroups

Variable	Single rater RC, degrees			Intra-rater RC for all raters, degrees		
	Rater 1	Rater 2	Rater 3	Pre-OP	Post-OP	Overall
T1–T12	5	11	9	9	9	9
T2–T12	6	12	13	12	9	11
T4–T12	5	7	10	9	6	8
T5–T12	5	7	10	10	5	8
T1-nonfixed	6	12	10	10	10	11
Nonfixed TK	6	12	13	12	10	11
L1–S1	6	6	7	9	12	11
Nonfixed LL	6	9	14	9	11	10

*RC* reliability coefficient, *TK* thoracic kyphosis, *LL* lumbar lordosis

**Table 3:** Inter-rater RC between three raters with pre- and postoperative subgroups

Variable	Inter-rater RC, degrees		
	Pre-OP	Post-OP	Overall
T1–T12	11	14	13
T2–T12	12	10	11
T4–T12	12	7	10
T5–T12	11	7	9
T1-nonfixed	12	14	13
Nonfixed TK	14	13	13
L1–S1	9	12	11
Nonfixed LL	11	12	11

*RC* reliability coefficient, *TK* thoracic kyphosis, *LL* lumbar lordosis

### Intra- and inter-rater reliability

**Fig. 2:**
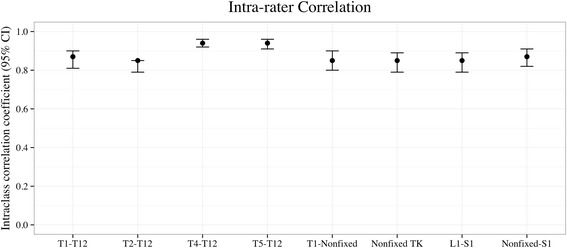
Intra-rater intra-class correlation coefficients for all measurements (both pre- and postoperative) with 95% confidence interval (*CI*). *TK* thoracic kyphosis

**Fig. 3:**
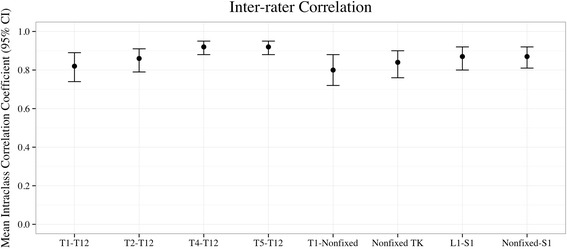
Inter-rater intra-class correlation coefficients of all measurements (both pre- and postoperative) with 95% confidence intervals (*CI*). *TK* thoracic kyphosis

Intra-rater ICC was highest for T4–T12 (0.94; 95% CI 0.92–0.96) and T5–T12 (0.94; 95% CI 0.91–0.96) and lowest for T2–T12 (0.84; 95% CI 0.79–0.85) (Fig. 2). Inter-rater ICC was highest for T4–T12 (0.92; 95% CI 0.88–0.95) and T5–T12 (0.92; 95% CI 0.88–0.95) and lowest for T1-nonfixed (0.80, 95% CI 0.72–0.88) (Fig. 3).

## Discussion

Our study noted a substantial measurement variation for all definitions of TK with the best reproducibility for T4–T12 and T5–T12 both in terms of intra- and inter-rater agreement as well as reliability. Only a few previous studies have addressed the variation of TK measurements in a systematic manner. For example, in a study by Ilharreborde et al. [35], the authors found an intra-rater agreement of 6° and 4° for T1–T12 and T4–T12, respectively, and an inter-rater agreement of 7° and 6°, respectively. This study, however, utilized EOS-imaging, which is a slot-scanning device that may improve the agreement. Moreover, EOS is currently only available in selective centers. Similarly, Kuklo et al. [36] found that the intra-rater agreement for T2–T12 and T5–T12 was 5° and 6°, respectively. However, none of these studies addressed the issue of random effects, so these results are not directly comparable to the present study and likely to underestimate the overall variation seen between randomly chosen raters. Carman et al. [37] measuring nonfixed TK found 95% of the differences between raters to be within 7° and found a trend towards less variation with increased clarity on radiographs. The study also found that an 11° difference in TK was required to rule out measurement error with 95% confidence. Our results are in line with these findings showing that TK measurements have considerable intra- and inter-rater variation and a difference of 8° to 13° (depending on TK definition) may solely be produced by observer error alone.

In order to ensure clinical applicability of our results, our study included both pre- and postoperative radiographs. Our analysis showed substantial differences in both intra- and inter-rater agreement between pre- and postoperative radiographs, showing markedly better agreement in postoperative radiographs for T4–T12 and T5–T12 (Tables 2 and 3). For the remaining TK measurements, analyses of pre- and postoperative subgroups were not conclusive but, generally, we found poorer or unchanged agreement. The reason for these changes may be that the variation seen in T4–12 and T5–T12 is mainly due to the lateral and rotational deformity of the curve which is surgically corrected whereas the variation seen in measurements including T1 and T2 is more likely due to structural overlap (e.g., of the humeral head) and therefore not affected by surgery. Interestingly, our analysis also showed considerable variation for the fixed and nonfixed LL, indicating that the sagittal radiograph, as a whole, is inherently difficult to analyze in a reproducible manner in AIS patients.

Our study focused on the overall TK because we found that a wide range of definitions exist in the literature. Establishing the respective reproducibility of these measurements was our main objective, but we would encourage future studies to include additional clinically important parameters, such as proximal TK (T2–T5) and thoracolumbar alignment (T10–L2) as well as several other clinically relevant measurements.

The ICC analysis showed good to excellent reliability for all measurements. However, while the ICC analysis is frequently reported in studies of this type, it holds limited practical value when assessing potential variation of individual measurements per subject, as it is a measure of the reliability for the measurement to differentiate between subjects. By applying a mixed effects model to our data, the observed variance is split into both the variability between the raters within subjects (inter-rater variation) and a residual error term (representing intra-rater variation). Ultimately, an RC is generated which represents the upper and lower 95% limits of agreement for an individual measurement. By using the rater as random effects, our results represent conservative estimates and we hypothesize that measurement variation found in our study would also apply for other raters.

Our results are limited by a substantial variation in single rater RC among raters, which was lowest for T4–T12 and highest for T2–T12 (Table 2). Several steps were taken before the initiation of the study to minimize bias in terms of discrepancies in labeling vertebra, handling of odd number of ribs, or definitions using the nonfixed approach. Rater 1 had more than 3 years of experience in evaluating radiographs from AIS patients, and raters 2 and 3 had 8 and 10 years of experience, respectively. It should be noted that all raters routinely use mainly T5–T12 or T2–12 when evaluating patients with AIS although rater 1 also uses the nonfixed approach on a regular basis. As such, we believe that our results reflect the expected variation between clinicians. In addition, our patient sample did not include lumbar curves, so we cannot infer that our results may be readily applied to this group. Also, the sample size in our study did not allow for analyzing individual curve types, but variation may be greater for thoracic curves since TK has been found to depend on curve type [38]. Nonetheless, we hope that our study can form the foundation whereby future studies can further elaborate upon different curve types.

Our results may guide clinicians and researchers in the evaluation of the sagittal profile following surgery in defining the limits of actual improvement of worsening of TK as opposed to expected measurement variation. Applying such variation in clinical definitions of progression has previously been described in guidelines for evaluation of radiographic results of brace treatment [37, 39, 40]. Our results indicate that T4–T12 and T5–T12 offer the least amount of observer variation, and while measuring nonfixed TK may offer a more individualized assessment of the spine, we found considerable measurement variation using this approach that may limit the clinical applicability. It is outside the scope of this paper to determine how these various measurements correlate with clinical outcomes; however, we recommend that future studies specifically state the applied definition of TK and also adequately address measurement variation when evaluating treatment results. This will further help with standardization of measurements between studies for comparative purposes.

## Conclusions

Our study addresses the intra- and inter-rater reproducibility of TK measurements in AIS patients, and we noted a considerable variation for all TK measurements. Both intra- and inter-rater reproducibility were best for T4–T12 and T5–12. Future studies should consider adopting a relevant minimum difference (depending on TK definition) as a limit for indication of true change in TK within a patient. As such, our findings have implications in the decision-making of the spine specialist.

## Additional file

Additional file 1: Original primary data. (XLSX 29 kb)

## Abbreviations

AIS: Adolescent idiopathic scoliosis
CI: Confidence interval
ICC: Intra-class correlation coefficient
LL: Lumbar lordosis
RC: Reliability coefficient
SD: Standard deviation
TK: Thoracic kyphosis
